# T2T reference genome assembly provides insights into anthocyanin accumulation in broccoli

**DOI:** 10.1093/hr/uhag110

**Published:** 2026-04-21

**Authors:** Chunqing Liu, Guangqing Li, Yuan Liu, Lei Huang, Jing Gong, Jian Pan, Jing Jiang, Xueqin Yao, Zhujie Xie

**Affiliations:** Shanghai Academy of Agricultural Sciences, Institute of Horticulture, Shanghai, China; Shanghai Academy of Agricultural Sciences, Institute of Horticulture, Shanghai, China; College of Horticulture, Shenyang Agricultural University, Shenyang, China; Shanghai Academy of Agricultural Sciences, Institute of Horticulture, Shanghai, China; Shanghai Academy of Agricultural Sciences, Institute of Horticulture, Shanghai, China; College of Horticulture, Shenyang Agricultural University, Shenyang, China; College of Horticulture, Shenyang Agricultural University, Shenyang, China; Shanghai Academy of Agricultural Sciences, Institute of Horticulture, Shanghai, China; Shanghai Academy of Agricultural Sciences, Institute of Horticulture, Shanghai, China

## Abstract

Broccoli (*Brassica oleracea* var. *italica*) is a widely cultivated cruciferous vegetable valued for its abundant bioactive compounds and nutraceutical properties. Among these, anthocyanins are not only important secondary metabolites contributing to nutritional and medicinal benefits, but also influence stress tolerance and the commercial quality of broccoli through purple pigmentation. However, the molecular mechanisms regulating anthocyanin biosynthesis in broccoli remain poorly understood, partly due to the incomplete genomic resources currently available. In this study, we constructed a telomere-to-telomere gap-free assembly of the broccoli genome using a combination of Oxford Nanopore Technology ultralong reads, PacBio high-fidelity reads, and Hi-C datasets. The resulting genome is 633.61 Mb in length, with an N50 of 60.36 Mb, and comprises gap-free assemblies of all 18 chromosomes, including complete telomere-to-telomere assemblies for nine chromosomes. Using this high-quality reference, we identified *BoF3’H*, a key gene regulating anthocyanin accumulation, which controlling the purple coloration of broccoli buds. To validate the function of the *BoF3’H* gene in anthocyanin biosynthesis, we used CRISPR-Cas9 gene editing to target and knock out the *BoF3’H* gene. The *bof3’h* mutant exhibited an 81.4% reduction in cyanidin and delphinidin derivative levels compared with those in the control. Metabolomic and transcriptomic profiling showed that the expression of 12 anthocyanin-related genes, including *PAL*, *C4H*, *CL3*, *CHS*, *F3’H*, and *ANS*, was downregulated. These findings elucidate the molecular basis of anthocyanin regulation in broccoli and provide a foundational genomic resource for evolutionary studies, gene discovery, and future breeding.

## Introduction

*Brassica oleracea*, the diploid species with a CC genome in the Triangle of U, is significant due to its morphological diversity and genetic proximity to other Brassica species, including broccoli (var. italica), cabbage (var. capitata), kale (var. acephala), cauliflower (var. botrytis), Brussels sprouts (var. gemmifera), and kohlrabi (var. gongylodes) [1]. Among the *B. oleracea* subspecies, broccoli is primarily cultivated for its edible flower buds and stalks [2]. In 2021, the global production of broccoli (combined with the production of cauliflowers) was 26 million tons, with China and India producing 72% of the global production quantity (https://en.wikipedia.org/wiki/Broccoli). Broccoli is popular because of its rich nutritional value and cancer-preventive properties; it contains high amounts of vitamin C, vitamin A, fiber, and sulforaphane [3].

Decoding complete genome sequences is essential for understanding the biological characteristics of plant species. The genome sequence of *B*. *oleracea* was first published in 2014 with 45 758 predicted genes and an N50 value of 21 kb [4, 5]. Recently, five improved *B. oleracea* genome sequences—Korso 1401, OX-heart 923, D134, C-8, and Cap02-12—were assembled using Pacific Biosciences (PacBio) HiFi long reads and chromosome conformation capture (Hi-C) data [6–10]. In the most recently published broccoli genome, one genotype, Bop04-28-6, was sequenced using a combination of PacBio HiFi reads and Hi-C technology, resulting in an updated contig N50 that reaches 14.7 Mb. However, some gaps and unanchored sequences remain, which comprise >6% of the total genome sequence length [11]. Recently, third-generation sequencing technologies have effectively avoided the difficulties in splicing repetitive sequences. Simultaneously, combining technologies such as Hi-C and Oxford Nanopore Technologies (ONT) to assist in genome assembly and filling in gaps (GAPs) can significantly improve the contig N50 metric and produce a telomere-to-telomere (T2T) genome [12]. T2T assemblies of various plant species, such as Arabidopsis, rice, maize, watermelon, bayberry, pods, soybean, carrot, strawberry, and sorghum, have been achieved [13–15]. However, to the best of our knowledge, no T2T genome/chromosome sequences in broccoli have been reported. The lack of high-quality genome assemblies limits the conduction of molecular genetic studies on broccoli. Therefore, it is crucial to de novo assemble a high-quality, near-complete broccoli genome and analyze the characteristics, structure, and distribution of its regions. This type of genome assembly will enhance the accuracy of genome assembly and provide a high-quality reference genome for a deeper understanding of the genes underlying critical agronomic traits.

The color of vegetables is a crucial commercial trait, and purple variants of cabbage, leaf mustard, broccoli, ornamental kale, and cauliflower; yellow and green variants of cauliflower; and white and orange flowering rapeseed are popular [16]. Broccoli heads are comprised of modified buds, and bud color is highly important for determining their commercial value. Broccoli buds can range in color from vibrant green to deep purple, and some green buds turn purple under abiotic stress environments, which influences the aesthetic appeal of such broccoli [17, 18]. Purple coloration occurs because of anthocyanin accumulation. Anthocyanins are a group of water-soluble pigments belonging to a larger class of flavonoids [19]. Flavonoids have numerous important physiological and biological functions in plants and humans. In addition to improved stress resistance, they may offer advantages in terms of promoting insect pollination and enhancing nutritional content [20]. Breeding new asparagus broccoli varieties with high anthocyanin content or no anthocyanin may enhance the economic value of vegetables [21]. Recent studies have identified several anthocyanin biosynthesis-related genes in brassica, including *BoMYB2*, *BoPAP1*, *BrF3H*, *BrMYBL2.1*, *BnTT1*, and *BnTT8* [16, 22–25]. F3’H and F3’5’H, belonging to the cytochrome P450 superfamily proteins CYP75B and CYP75A, respectively, have been linked with cyanidin and delphinidin biosynthesis and contribute to the purple and blue coloration of broccoli [20, 26]. However, in Brassicaceae species lacking CYP75A genes, only some CYP75B genes have been cloned and functionally characterized, including Arabidopsis F3’H (AtF3’H), B. rapa F3’H (BrF3’H), and B. napus F3’H (BnF3’H) [27–29]. Thus, further research is needed to reveal the genetic factors involved and enable breeding strategies targeting broccoli varieties that exhibit desirable bud characteristics.

In the present study, we performed T2T genome assembly of *B*. *italica* using an inbred line derived from the pot variety SN60. Through the integration of various genomic methods, we successfully performed map-based cloning of the candidate gene *BoF3’H* responsible for purple buds in broccoli. CRISPR/Cas9 technology was used to induce the targeted mutagenesis of the *BoF3’H* gene in broccoli. The significantly enhanced assembly of the broccoli genome presented in this study provides a crucial resource and establishes a strong foundation for functional genomic development and genetic improvement.

## Results

### Genome sequencing, assembly, and annotation

**Figure 1:**
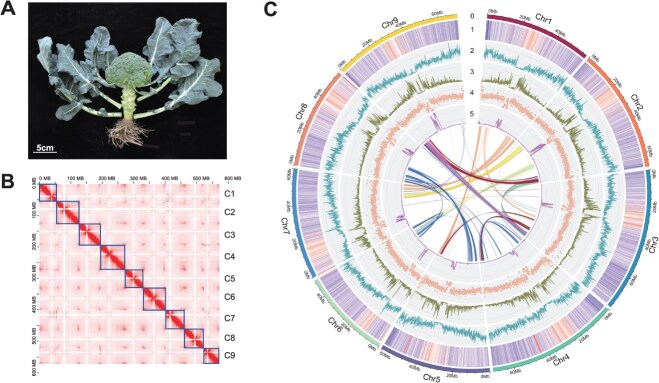
Telomere-to-telomere assembly of the SN60 genome. (A) Image showing key features of broccoli variety “SN60.” (B) Genome-wide Hi-C map of the SN60 genome . (C) Chromosome characterization of the SN60 genome. The outer layer of blocks represents nine chromosomes, and the marked regions indicate the centromeres.im The tracks from outside to inside indicate the following: chromosome length in megabases (Mb), GC content, gene density, Gypsy element density, TE density, and tandem repeat density.

The broccoli inbred line SN60, a highly homozygous genotype with early maturity and high yield traits, was selected for whole-genome sequencing. This line typically produces green buds (Fig. 1A). Multiple sequencing technologies, including Illumina sequencing, PacBio HiFi, ONT Ultralong, and Hi-C technologies, were used to assemble a high-quality genome for broccoli SN60. Using k-mer analysis of the Illumina reads (K = 17), a genome survey estimated the *B*. *italica* genome size to be 566 Mb, with a repetitive sequence rate of 67.2% (Fig. S1). Initially, approximately 39.13 Gb (~69.13× coverage of the genome) of PacBio HiFi reads and 85.23 Gb (~150.58× coverage of the genome) of ONT ultra-long reads were generated for genome assembly, with N50 values of 15.2 and 30.6 Mb in length, respectively (Table S1). To construct the chromosome-scale scaffolds, 162.24 Gb Hi-C data were used to order and orientate the contigs. As expected, the Hi-C interaction matrices displayed a distinct anti-diagonal pattern of intrachromosomal interactions (Fig. 1B). Subsequently, ONT and PacBio reads were aligned to the scaffolds to fill in the remaining GAPs. Finally, we achieved a 633.61-Mb gap-free genome of *B*. *italica,* containing nine chromosomes with a contig N50 length of 60.36 Mb. Our assembled version significantly improved several metrics compared with those of the previously released genomes BOP04-28-6 and HDEM, which were 613.79 and 545.12 Mb in length and had contig N50 values of 14.71 and 9.41 Mb, respectively (Table 1). Benchmarking universal single-copy orthologs (BUSCO) were used to evaluate genomic completeness; 99.4% of the core conserved plant genes were identified and completed, indicating the high quality of the assembled genome (Table S2). Collinearity analysis revealed a high degree of synteny between *B*. *italica* BOP04-28-6 and *B*. *italica* HDEM, confirming that the quality of the genome assembly was high (Figs S2 and S3). The existing reference genome HDEM contained 97 gaps, which were all filled in our assembly (Table 1).

**Table 1:** Comparison of genomic features of *B. oleracea* genome assemblies.

Genome features	This study	BOP04-28-6	HDEM
Length of the total genome, Mb	633.61	613.79	545.12
Number of contigs	850	552	269
Contig N50 length, Mb	60.36	14.71	9.49
Length of the chromosomes, Mb	572.93	576.18	528.86
Total size of unanchored contigs, Mb	60.68	37.62	16.26
Percentage of assembly in chromosome, %	90.42	93.87	95.27
Number of base chromosomes	9	9	9
Number of gap-free chromosomes	9	0	0
Number of gaps	0	75	97
Number of telomeres	18	17	16
Number of centromeres	9	9	9
TE size, %	58.19	58.85	58.64
GC content, %	36.96	36.62	36.01
Genome BUSCOs, %	99.4	99.40	97.01
LTR assembly index score	1	-	1.1
Gene number	58 044	55 958	61 279
Reference	This study	Wu *et al.* (2024)	Belser *et al.* (2018)

### Identifying the positions of telomeres and centromeres in chromosomes

**Figure 2:**
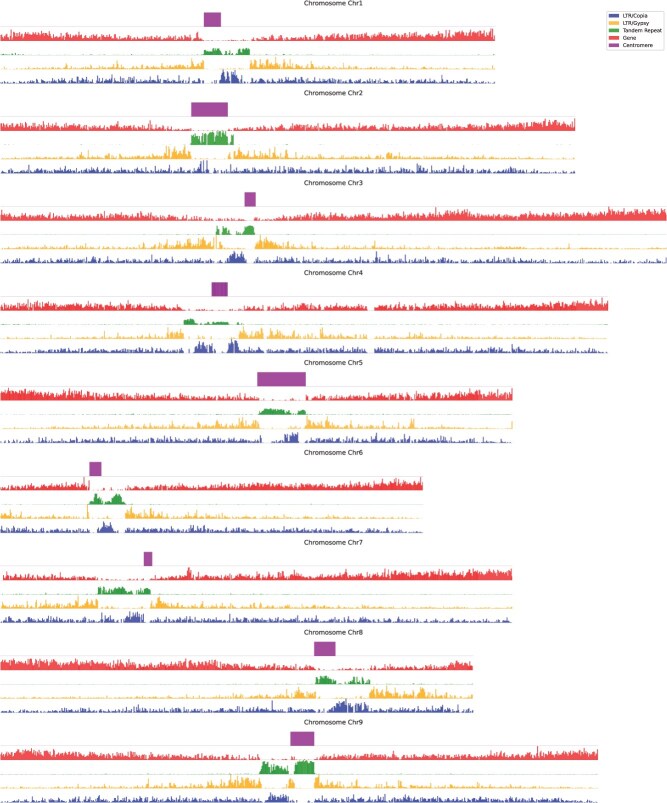
Delimiting of the SN60 centromeres.

By using the seven base telomere motif (“CCCTAAA” at the 5 end / “TTTAGGG” at the 3 end) as a sequence query, we successfully assembled all 18 potential telomeric regions with a maximum of 11 773 bp and a minimum of 118 bp and constructed nine T2T pseudomolecules for the SN60 genome (Table S3). The SN60 assembly included 18 telomeres, whereas no telomeres were identified in the BOP04-28-6 genome, and 16 telomeres were identified in the HDEM genomes. For centromeric localization, we scanned candidate repeat sequences using the Tandem Repeats Finder (TRF) and identified each centromeric sequence, with the lengths of the centromeres ranging from 1.09 to 5.72 Mb (Fig. 2). In the centromeric region of SN60 T2T, 132 candidate genes were identified. Kyoto Encyclopedia of Genes and Genomes (KEGG) functional enrichment analysis indicated that these genes were mainly related to “Basal transcription factors,” “Cyanoamino acid metabolism,” “Starch and sucrose metabolism,” “Inositol phosphate metabolism,” “Phosphatidylinositol signaling system,” and “Phagosome” and “Autophagy” (Table S4, Fig. S4).

### Genome annotation analysis

The results of repetitive sequence annotation, conducted using a combination of de novo and homology-based methods, showed that 58.19% of the broccoli genome contained repeat sequences with a size of 333.39 Mb. These were predominantly long-terminal repeat (LTR) elements (32.74%), followed by TIR (12.33%), long interspersed nuclear elements (LINE) (2.63%), and short interspersed nuclear elements (0.54%) (Table S5). For annotation of the broccoli genome, an integrated analysis involving de novo prediction, homology-based searches, and RNA sequencing was used to predict gene structures. As a result, 58 044 genes encoding proteins were predicted and annotated by homology proteins, domains, or expressed transcripts, with an average gene length of 1945.6 bp and an average CDS length of 252.9 bp (Table 1; Fig. 2; Fig. S5). Among these genes, 58 044 were annotated using multiple protein-related databases, with 54 930 (94.64%) genes identified in eggNOG, 51 613 (88.92%) genes in COG/KOG, 49261 (84.87%) genes in PFAMS, 38745 (66.75%) genes in Uniport, 25 494 (43.92%) genes in GO, and 16 161 (27.84%) genes in KEGG (Table S6). Specifically, 27 663 transcription factors (TFs) were identified in the *B*. *italica* genome and classified into 54 TF families consisting of 4586, 3776, 2312, 2213, 2108, and 1170 of MYB, ERF, bHLH, C2H2, Dof, and WRKY, respectively (Table S7). In addition, we identified various noncoding RNA, including 75 560 miRNAs, 1441 tRNAs, 2990 rRNAs, and 3445 snRNAs (Table S8).

### Comparative genomic and genome evolution analysis

**Figure 3:**
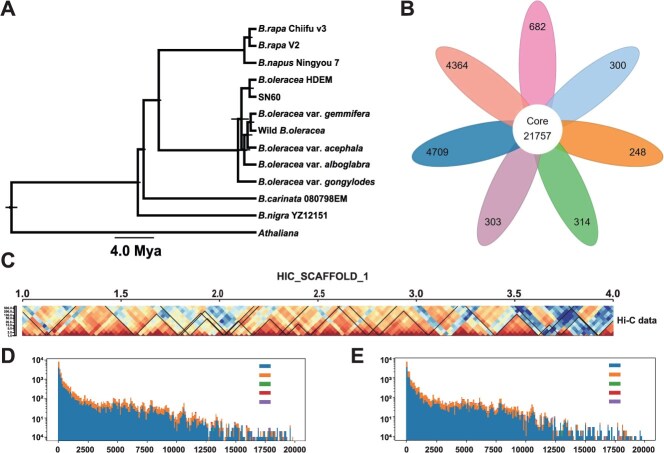
Comparative genomic analyses. (A) Phylogenetic tree of 13 plant species. (B) Venn diagram of the orthogroups across seven Brassicaceae genomes. (C) 3D chromatin structure of the SN60 genome. (D) SV length analysis between SN60 and BOP04-28-6. (E) SV length analysis between SN60 and HDEM.

To elucidate the pathway of broccoli genome evolution, 13 sequenced cruciferae genomes were subjected to phylogenetic analysis, including one *Arabidopsis thaliana*, one *B. carinata*, one *B. napus*, two *B. nigra* (Ni100 and YZ12151), two *B. rapa* (Chiifu and Z1), and six *B. oleracea* (T08, T09, T11, T18, T27, and HDEM) (Table S9) [30, 31]. Phylogenetic analysis based on the 32 906 shared single-copy orthologous gene families indicated that *B. rapa* and *B. oleracea* diverged from *A. thaliana* at approximately 4.0 MYA while 14 885 and 800 gene families showed significant expansion and contraction in broccoli, respectively (*P* < 0.01) (Fig. 3A). Additionally, phylogenetic analysis showed that the AA (*B. rapa* chifu v3 and *B. rapa* Z1), BB (*B. nigra* Ni100), and CC genomes (*B. oleracea var. Alboglabra, B. oleracea var.* italica HDEM, *B. oleracea* var. Gemmifera, Wild *B. oleracea*, *B. oleracea* var. Gongylodes, and Bol SN) were divided into different branches, and *B. napus* Ningyou 7 was located in the AA genome branch. Species with the CC genome were more closely positioned to those with the AA genome than to those with the BB genome. Among the different *B. oleracea* species, the floret balls (SN60 and HDEM) diverged from the leaf balls (*B. oleracea* var. acephala, OX-heart 923) and *B. oleracea* var. gongylodes almost simultaneously (Fig. 3A). The expanded gene families were significantly associated with the following GO enrichment terms: macromolecules and cellular macromolecules in the biological process category (*P* < 0.05, FDR <0.05; Fig. S6). Additionally, 21 757 gene families were shared between *A. thaliana, B. rapa*, and *B. oleracea*, whereas 682 genes were specific to *B. italica* (Fig. 3B). Most broccoli-specific genes were enriched in stress responses, such as response to stimuli, response to stress, and defense responses (Fig. S7).

We further investigated the prevalence of single-nucleotide polymorphisms (SNPs), insertions/deletions (InDels), and structural variants (SVs) by comparing the SN genome with that of two other broccoli species, BOP04-28-6 and HDEM. In total, 2 243 422 SNPs/InDels and 26 977 SVs were identified across SN and BOP04-28-6 (Fig. 3D, Tables S10 and S11). In contrast, 2 165 416 SNPs/InDels and 21 985 SVs were identified between SN60 and HDEM (Fig. 3E, Tables S12, and S13). There were more alterations in deletions, insertions, and SVs between SN60 and BOP04-28-6 than between SN60 and HDEM. Between SN60 and BOP04-28-6, the indel mutations involved 2246 genes and the SVs corresponded to 2118 genes, which were functionally enriched in organophosphate ester transport, positive regulation of amide metabolic process, obsolete cell projection part, and cellular response to osmotic stress, as determined by GO analyses (Figs S8 and S9). Between SN60 and HDEM, 2598 and 1948 genes were associated with InDels and large SVs, respectively, among which 671 and 598 genes were functionally enriched according to the results of the GO analysis (Figs S10 and S11). These genes provide valuable resources for broccoli breeding. The Hi-C data visualization map shows the interaction patterns of the chromosomes, which is highly important for understanding biological processes such as gene regulation and chromosome interactions (Fig. 3C).

### Identification of gene expression in purple pods using the SN60 genome

**Figure 4:**
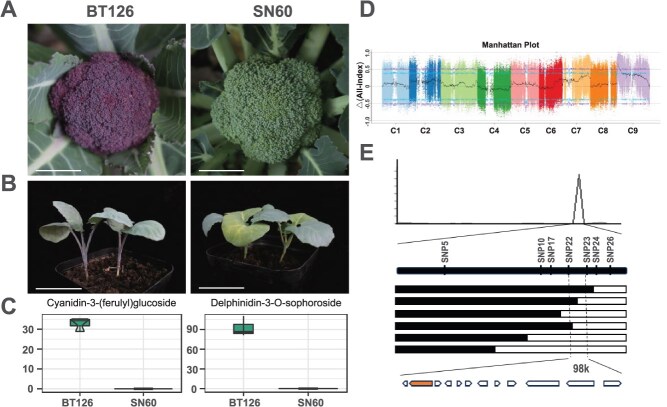
QTL analysis of plants with contrasting pigmentation phenotypes. (A) Phenotype of bud heads in BT126 and SN60. Bars, 5 cm. (B) Phenotype of seedlings in BT126 and SN60. (C) Analysis of anthocyanidin contents using HPLC. Data are presented as mean ± standard deviation (SD) of six independent biological replicates. (D) Distribution of Δ SNP index on chromosomes. (E) Fine mapping of *BoPur* to a 73-kb region on chromosome 09.

To determine the genes associated with purple coloration in pods, we conducted a genetic analysis using reciprocal hybridization between the BT 126 (purple pods and seedlings) and SN60 (green pods and seedlings) varieties (Fig. 4A and B). A total of 302 F2 individuals were obtained, comprising 33, 52, 75, 79, and 63 individuals with purple, slightly darker purple, light purple, slightly purple, and green pods, respectively. Cyanidin and pelargonidin were detected in the purple pods using HPLC analysis. Additionally, anthocyanidin levels were low in the green pod broccoli (Fig. 4C, Table S14). To detect the *BoPur* genes responsible for broccoli purple pods, we sequenced the two parents as well as two bulked pools with extreme phenotypes from 30 F2 individuals per pod color. Bulk segregant analysis (BSA) identified a locus on chromosome C09 using the SN60 genome as a reference. By investigating the variant number with a SNP-index >0.9 using the 1 kb sliding window method, we identified a genomic region spanning 6.38 Mb (3.10–3.65 Mb) (Fig. 4D). To narrow down the mapping region and isolate the candidate gene underlying the *BoPur* locus, 31 markers were developed based on the SNPs identified in the two parents aligned to the SN60 reference genome. Fourteen SNP markers were selected for the screening of 302 F2 mapping populations. The T2T assembly was instrumental in the precise identification and functional validation of the key candidate gene. The gap-free sequence of chromosome C09 enabled us to unambiguously narrow the *BoPur* locus to a 73-kb interval and identify *HiC_scaffold9_3907674* as the sole candidate gene encoding flavonoid 3′-hydroxylase (Fig. 4E). Gene-specific primers were designed to amplify and sequence candidate genes from the genomic DNA fragments of the parents (Table S15). The sequence alignment of the two parent genomes revealed a 43-bp deletion in the second exon of *BoF3’H*, which created a premature stop codon and significantly decreased *BoF3’H* mRNA levels in the green parent SN60 (Fig. S12). To further validate the association between the premature stop codon in *BoF3’H* and head color phenotype, a segregating F₂ population was employed for genetic verification. Based on the InDel polymorphism responsible for the premature stop codon in BoF3’H, an InDel molecular marker *BoF3’H*-1F/1R (forward: 5′- CACGGACATGCTTAGCACTTTAAT-3′; reverse: 5′- GCCGTAAACATGTTCTGTAATGGA) was developed and applied to genotype 200 F₂ individuals. PCR amplification revealed two distinct fragment sizes, 187 and 144 bp. All individuals displaying green buds produced only the 144 bp fragment, whereas purple-bud individuals showed either a single 187 bp fragment or both 187 and 144 bp fragments, corresponding to homozygous wild-type and heterozygous genotypes, respectively (Fig. S13).

**Figure 5:**
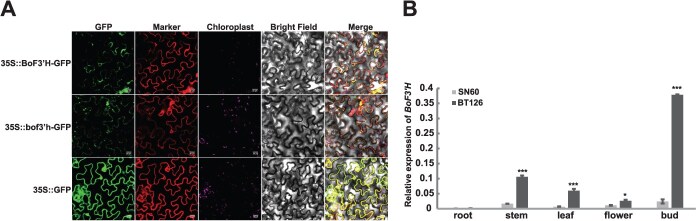
Subcellular localization and qRT-PCR of BT126 and SN60. (A) The subcellular localization of *BoF3’H* and *bof3’h*. (B) qRT-PCR analysis of *BoF3’H* in different organs of BT126 and SN60. Data are presented as mean ± SD of three independent biological replicates. Significant differences were determined by Student’s *t*-test (*P* < 0.05).

To elucidate the expression pattern of *BoF3’H*, we conducted a subcellular localization and qRT-PCR analysis. qRT-PCR analysis revealed that the expression level of *BoF3’H* was significantly higher in the purple broccoli BT126 compared to the green broccoli cultivar SN60. Notably, *BoF3’H* exhibited the highest expression in flower buds, followed by leaves and stems, with no detectable expression in roots (Fig. 5B). To further investigate the subcellular localization of the BoF3’H protein, a transient expression assay was conducted in tobacco leaves using a BoF3’H-GFP fusion protein. The results demonstrated that BoF3’H-GFP localized predominantly in the endoplasmic reticulum. However, the localization of the *bof3’h*-GFP mutant was aberrant, with uneven distribution in the endoplasmic reticulum, weaker fluorescence intensity, and the formation of granular aggregates (Fig. 5A). This likely resulted from a truncated translation sequence that altered the protein structure, leading to ubiquitination-mediated degradation, which prevented the formation of stable localization.

**Figure 6:**
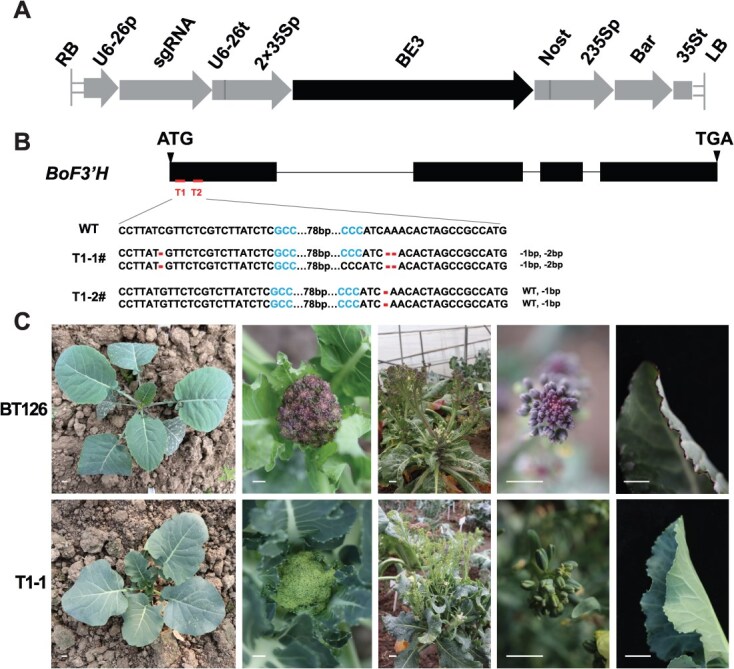
CRISPR/Cas9-induced *BoF3’H* gene mutants and their phenotypes. (A) Schematic diagram of the CRISPR/Cas9 construct targeting *BoF3’H*. (B) Schematic of the *BoF3’H* gene structure and sgRNA targeted sequences from the wild-type (WT) and representative mutants T1–1# and T1–2#. (C) Phenotypes of the wild-type and *bof3’h* mutant T1–1. Data are presented as mean ± SD of three independent biological replicates. Bars, 1 cm.

To validate the candidacy of the *BoF3’H* gene, we employed the CRISPR/Cas9 system to generate a knockout in BT 126. Two sgRNA sequences containing protospacer adjacent motifs (PAM: NGG) were selected using the CRISPOR tool (http://crispor.tefor.net/). The complete gene model, devoid of assembly artifacts, was critical for accurate sgRNA design for CRISPR-Cas9 targeting within the first exon (Fig. 6A and B). The assembled CRISPR-*BoF3’H* vector was successfully transformed into broccoli BT 126, resulting in the emergence of transgenic lines displaying varying levels of purple coloration (Fig. 6C). To obtain genetically stable mutant lines, two independent T0 *bof3’h* CRISPR/Cas9-edited plants were self-pollinated to generate T1 progeny populations. Target-site mutations in T1 individuals were systematically analyzed using high-throughput tracking of mutations (Hi-TOM) sequencing. A total of seventeen independent T1 lines carrying homozygous mutations in *BoF3’H* were identified. Among them, six lines harbored homozygous mutations at the sgRNA-1 target site, while eleven lines contained homozygous mutations simultaneously at both sgRNA-1 and sgRNA-2 target sites (Fig. 6b). Sequence analysis indicated that all detected homozygous mutations caused frameshift insertions or deletions, which were predicted to generate truncated, nonfunctional BoF3’H proteins. Importantly, the homozygous mutations were stably inherited in the T1 generation and showed clear genotype–phenotype consistency. All T1 plants carrying homozygous *bof3’h* mutations exhibited green buds phenotype, consistent with the reduced anthocyanin accumulation observed in T0 plants (Fig. 3c). These results demonstrate the genetic stability and heritable nature of the CRISPR/Cas9-induced *bof3’h* mutations.

Anthocyanin levels in wild-type and *bof3’h* plants were further analyzed using UPLC–MS. Naringenin, quercetin, cyanidin, delphinidin, pelargonidin, petunidin, malvidin, and peonidin contents were measured in both the wild-type BT 126 and *bof3’h* mutant. Notably, the *bof3’h* mutant showed a significant reduction in cyanidin (by 64.1%) and delphinidin (by 97.2%) content, and the total amount of anthocyanin was 81.4% less than that in the wild-type (Fig. 6C, Table S14). Therefore, we inferred that *BoF3’H* plays an essential role in anthocyanin biosynthesis in broccoli.

**Figure 7:**
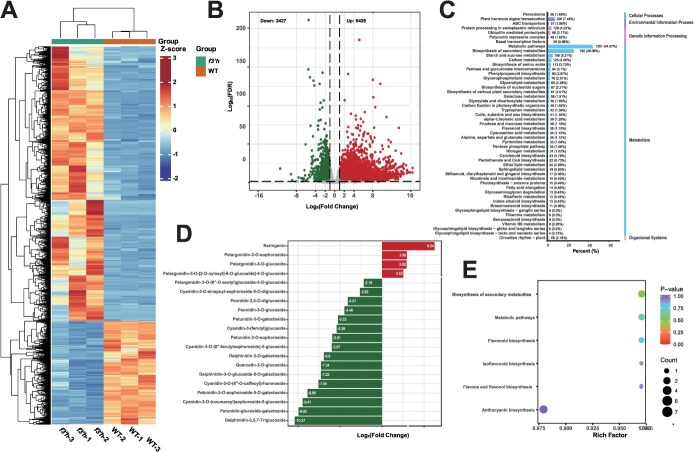
Transcriptome and metabolome analysis revealed the pivotal role of *BoF3’H* in wild-type and *bof3’h* mutant. (A) Gene expression clustering analysis. (B) RNA-seq differential gene expression volcano plot. DEGs were identified using DESeq2 with an adjusted *P*-value (False Discovery Rate, FDR) < 0.05 and |log₂(fold change)| > 1 (dashed lines). Each point represents a gene, based on three biological replicates per genotype. (C) Bar graph of KEGG pathway enrichment analysis of differentially expressed genes. The rich factor and -log₁₀(*P*-value) are shown. Enrichment significance was determined by hypergeometric test with FDR correction (Q-value < 0.05). (D) Differential metabolites bar chart. (E) KEGG pathway enrichment of differential metabolites. Data are presented as mean ± SD of six independent biological replicates. Significant differences between genotypes for each tissue were assessed by Student’s *t*-test (*P* < 0.05).

Furthermore, to elucidate how *BoF3’H* affects anthocyanin biosynthesis in broccoli, we analyzed gene expression levels in the BT 126 and *bof3’h* mutant using RNA-seq. There were 6426 and 2427 genes with upregulated and downregulated expression, respectively, between BT 126 and the *bof3’h* mutant (Fig. 7A and B). KEGG analysis revealed a significant enrichment of terms associated with plant hormone signal transduction, flavonoid biosynthesis, and phenylpropanoid biosynthesis (Fig. 7C). Compared with those in BT 126, the levels of the differentially expressed genes (DEGs) associated with anthocyanin biosynthesis (*PAL*, *C4H*, *CL3*, *CHS*, *F3H*, *F3’H*, *FLS*, *UGT78D2*, *MYB12*, *PAP1*, *PAP2*, *ANS*, and *EGL3*) were decreased in the *bof3’h* mutant. Transcriptome and metabolome association analyses showed that the DEGs involved in anthocyanin, isoflavonoid, and flavonoid biosynthesis accounted for a relatively high proportion of the total gene expression (Fig. 7D and E). In summary, the *bof3’h* mutation affected the expression transcript levels of key genes related to anthocyanin biosynthesis. In addition to the DEGs associated with anthocyanin biosynthesis, we conducted a comprehensive functional analysis of the 6426 upregulated genes identified in purple buds. GO enrichment analysis showed that these genes were significantly enriched in biological processes related to abiotic stress responses, including “response to oxygen levels,” “response to reactive oxygen species,” and MAPK signaling components. KEGG pathway enrichment further revealed overrepresentation of peroxisome and protein processing in endoplasmic reticulum. Moreover, a substantial number of upregulated genes were associated with hormone signaling pathways, particularly auxin, abscisic acid (ABA), and jasmonic acid (JA), indicating activation of hormone-mediated regulatory networks. The enrichment of these functional categories indicates that purple bud formation in broccoli is accompanied by activation of multiple stress- and hormone-responsive pathways, which may interact with flavonoid regulatory networks and potentially influence anthocyanin accumulation.

## Discussion

High-quality genome assembly at the chromosome level, with a near-complete, highly contiguous, and well-organized reference genome, can improve our understanding of the molecular causes of economically important traits in plants and provide a scientific basis for crop improvement [32, 33]. The rapid advancement of long-read sequencing technologies for ultra-long ONT sequencing and PacBio HiFi has provided powerful tools for generating high-quality genome assemblies. Hi-C technology is a powerful tool for assembling fragmented plant genomes at the chromosomal scale [12]. Recently, two *B. italica* genomes have been assembled; however, many sequences have not been assembled [11, 34]. To overcome the inherent challenges of assembling the highly repetitive (~67.2%) and structurally complex *B. oleracea* genome, we employed a synergistic multiplatform strategy: ONT ultralong reads (N50 = 30.6 Mb) provided the necessary length to span massive centromeric satellite arrays and telomeric repeats; PacBio HiFi reads (69.13 × coverage) delivered single-molecule accuracy for base-level polishing, critical for resolving homopolymer errors in coding and regulatory regions; Hi-C data anchored contigs into chromosome-scale scaffolds while resolving topological ambiguities arising from ancestral genome triplication. This integrated approach enabled the first T2T assembly of broccoli, encompassing all 18 telomeres and 9 complete centromeres (ranging 1.09–5.72 Mb) with unprecedented continuity (contig N50 = 60.36 Mb). Compared to the best previous assembly (BOP04-28-6, contig N50 = 14.71 Mb), our assembly reduced fragmentation by 4-fold and completely eliminated 97 gaps present in HDEM. Crucially, this gapless assembly allowed us to fully characterize pericentromeric and subtelomeric regions, which are often misassembled or collapsed in draft genomes. For instance, we precisely delineated all nine centromeres, ranging from 1.09 to 5.72 Mb, and identified 132 candidate genes embedded within these centromeric repeats (Fig. 2, Table S4). KEGG enrichment analysis revealed these genes are involved in fundamental processes such as “Basal transcription factors,” “Inositol phosphate metabolism,” and “Phosphatidylinositol signaling system” (Fig. S4), suggesting potential roles in centromere function and chromosome segregation beyond their canonical annotations. Furthermore, the complete resolution of all 18 telomeres (Table S3) provides a definitive reference for studying telomere biology, end-replication problems, and the dynamics of subtelomeric regions, which are often hotspots for disease resistance and adaptive gene families in plants. This structural completeness transforms these regions from “gaps” into accessible targets for functional genomics. This T2T resource provides a complete genomic landscape, which is essential for precise gene editing (CRISPR targeting of *BoF3’H* exon 1) and accurate interpretation of regulatory elements governing traits like bud color [11, 34].

In addition, comparative genomics of the two sequenced broccoli genomes, HDEM and BOP04-28-6, was performed, and the presence of three types of mutations (SNPs, indels, and SVs) was characterized. The completeness of our assembly provides an authoritative framework for interpreting genetic diversity. Comparative analyses with genomes BOP04-28-6 and HDEM revealed over 2.2 million SNPs/InDels and ~26 977 SVs (Fig. 3D and E, Tables S10–S13). Notably, the T2T assembly allows for the accurate mapping of large SVs, particularly in repetitive centromeric and telomeric regions, which are often mischaracterized. GO enrichment of genes affected by SVs and InDels between SN60 and BOP04-28-6 highlighted terms like “cellular response to osmotic stress” and “organophosphate ester transport” (Figs S8 and S9). This suggests that structural variation, now fully resolvable, may be a key driver of adaptive traits such as stress tolerance. For breeding, this genome serves as an essential resource for marker-assisted selection and genome editing. The identification of broccoli-specific gene families enriched in “response to stimuli” and “defense responses” (Figs 3B and S7), along with a comprehensive catalog of transcription factors (54 families, 27 663 TFs, Table S7), pinpoints direct targets for engineering improved anthocyanin content and resilience.

As expected, SN had significantly higher numbers of SNPs/indels (2 243 422) and SVs (26 977) compared with BOP04-28-6 than when compared with HDEM. SVs consist of deletions and insertions that contribute to genetic diversity and are important factors in evolution; however, they also pose risks by altering gene function, which may be potentially harmful [35]. Characterization of genetic variations across species, subspecies, and varieties can reveal the underlying genetic architecture that contributes to essential traits, such as disease resistance, stress tolerance, yield, and nutritional value [7, 8, 30, 36]. Importantly, the interpretation of these genomic variations and structural features greatly benefits from publicly available Brassicaceae omics resources. Databases such as the Brassica Database (BRAD; http://brassicadb.cn/), which provides comprehensive genome annotations, synteny relationships, and genetic variation data for multiple Brassica species, and the ten Brassicaceae Genomes Resource (TBGR; http://www.tbgr.org.cn), which integrates high-quality genome assemblies and multi-omics datasets across key Brassicaceae taxa, offer essential comparative frameworks. By integrating our T2T broccoli genome with these databases, we further enhance the resolution of evolutionary inference and functional genomics analyses, enabling more accurate identification of orthologous gene families, SVs, and lineage-specific expansions associated with phenotypic diversification and plant evolution.

Anthocyanins, including cyanidin, pelargonidin, and delphinidin derivatives, exhibit diverse coloration in plant tissues ranging from pale yellow to deep violet. In Brassica species, purple pigmentation predominantly results from cyanidin accumulation, as documented in cabbage, kale, and purple cauliflower [37, 38]. Our study corroborates these findings, with LC–MS analysis revealing cyanidin and delphinidin as the major pigments in purple broccoli buds (Fig. 4). Notably, Wen *et al.* reported malvidin in broccoli flower buds, but our metabolite profiling detected cyanidin and delphinidin, suggesting potential cultivar-specific differences in anthocyanin composition [20]. To further investigate whether the functional differences of *BoF3’H* were associated with cis-regulatory divergence, we sequenced and compared the 2-kb promoter regions of BoF3’H from SN60 and the green-bud genotype WT-G. Promoter alignment revealed multiple polymorphisms, including 12 InDels and several SNP clusters. Cis-element analysis using PlantCARE indicated that *BoF3’H*-BT126, *Bol043829* and *BnaC09G0570900ZS* harbors additional MYC, G-box, ERE, ABRE and light-responsive elements, which are absent or reduced in BraA10g02723P and BnaA10G0256900ZS. These variations coincide with the significantly higher *BoF3’H* expression levels observed in BT126, suggesting that promoter structural divergence likely contributes to the altered transcriptional regulation and anthocyanin biosynthesis differences between different cultivars. This evidence strengthens our conclusion that *BoF3’H*’s functional divergence is at least partly driven by cis-regulatory variation rather than coding-sequence changes alone. To clarify whether this discrepancy resulted from biological differences or methodological artifacts, we conducted comprehensive method validation experiments, including standard calibration, dual-mode ionization tests, gradient extension, and optimization of MRM transitions. These assays confirmed that our UPLC–MS conditions provided sufficient sensitivity and chromatographic resolution for detecting malvidin when present. Therefore, the absence of malvidin in BT126 is unlikely due to analytical constraints. Combined with the documented diversity of anthocyanin biosynthetic flux among *B. oleracea* cultivars, our findings support the interpretation that anthocyanin composition in broccoli is genotype-dependent, with SN60 favoring the cyanidin and delphinidin branches. This pattern is consistent with the elevated expression of *F3’H* and *F3’5’H* pathway genes observed in our transcriptome analyses, which may redirect metabolic flux away from the 5′-hydroxylated pathway required for malvidin biosynthesis. Together, these results underscore the substantial metabolic variation among broccoli germplasms and highlight the importance of integrating metabolomic and genetic evidence to interpret anthocyanin diversity.

In previous studies, one major single locus and two minor loci for anthocyanin accumulation in broccoli sepals were identified on chr01, accounting for 9.6%–16.5% of the phenotypic variation [39]. Through QTL mapping using the SN60 genome, we identified a 73-kb locus on chromosome C09 (51.71–51.78 Mb) associated with anthocyanin biosynthesis (Fig. 4A–C). Within this interval, *HiC_scaffold9_3907674 e*merged as the prime candidate due to its homology to *F3’H* genes. Furthermore, comparative analysis revealed that *BoF3’H* shares 92% amino acid identity with *BrF3’H* from purple Chinese cabbage but regulates bud-specific—rather than leaf-specific—pigmentation. This functional divergence, now discernible with precise genomic context, may be attributable to differences in cis-regulatory elements in their promoters, a hypothesis that can be directly tested using our assembly as a reference [18, 40].

The CRISPR-Cas9-mediated knockout of *BoF3’H* resulted in an 81.4% reduction in total anthocyanin content and a complete loss of purple pigmentation (Fig. 6C), confirming its pivotal role through loss-of-function evidence. This also demonstrates how a complete genome transforms candidate gene prediction into confirmatory functional genetics. It should be noted that while the knockout phenotype strongly supports the necessity of *BoF3’H* for anthocyanin accumulation, complementation of the *BoF3’H* allele would provide the most direct causal proof. Such experiments were not completed due to the long generation time and transformation cycle of broccoli. Nevertheless, the concurrent evidence from fine-mapping (Fig. 4E), the natural 43-bp deletion allele in green cultivars (Fig. S12), its specific expression pattern in pigmented tissues (Fig. 5B), and the coordinated downregulation of the entire anthocyanin pathway in the *bof3’h* mutant (Fig. 7) collectively provide robust support for *BoF3’H* as the key regulator at the *BoPur* locus. This aligns with Jung *et al.*’s report on *F3’H* function in flavonoid biosynthesis (e.g., 94% reduction in purple rice) [41]. Our findings not only resolve the paradoxical co-occurrence of cyanidin and delphinidin derivatives in broccoli flower buds—previously attributed solely to *F3’5’H* activity—but also provide mechanistic evidence for leveraging *BoF3’H* in breeding programs to enhance anthocyanin diversity [20]. Beyond the downregulation of key anthocyanin biosynthetic genes, our transcriptomic data revealed substantial activation of stress- and hormone-related pathways during purple bud formation. The 6426 upregulated genes were enriched in ROS-detoxification pathways, MAPK signaling modules, and multiple hormone-signaling branches, including auxin, ABA, and JA. These results suggest that anthocyanin accumulation in broccoli may not occur in isolation but rather in coordination with broader physiological transitions involving stress mitigation and hormonal regulation. Previous studies have shown that ROS homeostasis, MAPK signaling, and hormone crosstalk can modulate MYB–bHLH–WD40 (MBW) complexes, thereby influencing anthocyanin biosynthesis [19, 27, 36]. Thus, the enrichment of these pathways in our dataset supports the hypothesis that purple coloration in SN60 is associated with integrated transcriptional reprogramming affecting both primary stress responses and secondary metabolism. Future studies should explore the epistatic interactions between *BoF3’H* and upstream transcription factors, which could elucidate combinatorial strategies for metabolic engineering in Brassica species.

In summary, beyond technical metrics, this first T2T genome of broccoli delivers substantial biological novelty: it unveils the complete architecture of centromeres and telomeres with associated genes, enables the precise mapping and causal validation of trait-governing genes like *BoF3’H* via genome editing, and provides an unambiguous reference for exploiting structural variation and lineage-specific genes in evolutionary studies and molecular breeding programs aimed at enhancing vegetable quality and stress adaptation.

## Materials and methods

### Plant materials and whole-genome sequencing

The elite inbred line of *B. oleracea* L var. *italica* (SN60, broccoli) was obtained from the Zhuanghang Experimental Station of the Shanghai Academy of Agriculture Sciences, Shanghai, China. This line is characterized by high homozygosity, early maturity, and high yield potential. The days to maturity for SN60 were observed to be approximately 60–65 days after transplanting, and it produced an average yield of 450–500 grams per plant under standard cultivation conditions. Whole-genome DNA sequences were generated using a combination of the Illumina, ONT, and PacBio HiFi platforms. Genomic DNA was extracted from young leaves using cetyltrimethylammonium bromide (CTAB). The integrity of the extracted DNA was assessed using 1.0% agarose gel electrophoresis, and the concentration and purity of the DNA were determined using a NanoDropTM D-1000 spectrophotometer (NanoDrop Technologies, Wilmington, DE, USA). Illumina sequencing libraries were constructed on an Illumina HiSeq X platform according to the manufacturer’s standard protocol, with the insert fragment size set to 300 bp. Then, we constructed the Oxford nanopore sequencing library using the Oxford Nanopore Technologies SQK-LSK109 genome sequencing kit (SQK-LSK109). Sequencing was performed on the PromethION platform, and high-precision base calling was performed using the Guppy software (version 4.0.15). For SMRT sequencing, 50 mg of DNA was obtained to prepare a SMRTbell library using the SMRTbell Express Template Prep Kit 2.0 (Pacific Biosciences, CA, USA). We utilized Hi-C sequencing technology for genome scaffolding. Cross-linked DNA was isolated, purified, and digested with DpnII, followed by blunt-end repair and biotin tagging. Biotin-labeled DNA fragments were captured and enriched using PCR to construct the Hi-C library. Sequencing was performed on an Illumina HiSeq X platform using the PE150 strategy, which enables paired-end reads for high-resolution structural information. RNA samples from the root, stem, leaf, and head tissues were prepared according to the manufacturer’s protocols, and their quality and quantity were assessed using a NanoDrop spectrophotometer, Qubit 3.0 Fluorometer, and Agilent Bioanalyzer 2100. Paired-end libraries with 300 bp insert sizes were constructed using the TruSeq Sample Preparation Kit (Illumina Inc., San Diego, CA) and sequenced on the Illumina HiSeq X platform, facilitating high-throughput RNA sequencing.

### Genome survey, assembly, and quality assessment

The genome size and characteristics were estimated using a k-mer-based approach. K-mers of 21 base pairs (bp) in length were generated using the JELLYFISH software, a tool designed for efficient k-mer counting. Subsequently, genome characteristics, including genome size, proportion of repetitive sequences, and heterozygosity levels, were analyzed and estimated using the GENOMESCOPE software. ONT long reads were subjected to de novo assembly using NEXTDENOVO (v2.0, GitHub Link) with the default parameters. The initial assembly was polished over three iterative rounds using ONT and Illumina reads in NEXTPOLISH (v1.0). Kilobase-resolution Hi-C data and maps were processed from FASTQ raw data files using the Juicer platform. Scaffolds were constructed from polished contigs using a 3D-DNA pipeline (v180922). The completeness of the genome assembly was evaluated using BUSCO (v4.0.5). The read-mapping ratio was calculated by mapping ONT long reads and Illumina reads to the genome assembly using MINIMAP2 (v2.24). Assembly contiguity was assessed using the LAI derived from the outputs of LTR_RETRIEVER. Comparative genomic analysis of the present and recently published chicory genomes was conducted. Pairwise synteny analysis was performed using the JCVI software. SNPs and SVs between the two genomes were identified using SAMTOOLS and DELLY, respectively.

### Gene annotation

To identify the repeat sequences in the broccoli genome, a nonredundant repeat sequence library was constructed using the EDTA pipeline (v1.9.4). Genome annotation was performed using the MAKER-P pipeline (v2.31.8), utilizing three approaches: ab initio prediction, homology-based prediction, and RNA-Seq-based prediction.

Protein sequences from six species in the Brassica family and 1440 orthologs of embryophytes were collected using the BUSCO software (v5.2.2). Transcripts obtained from the de novo assembly were used to train the gene prediction parameters for SNAP (v2006-07-28) and AUGUSTUS (v3.2.2). Gene functions were assigned by evaluating their homology with known proteins. The predicted gene sequences were compared to those in the NCBI nonredundant protein database and the UniProtKB Swiss-Prot protein database using BLASTP.

### Phylogenetic analysis

To investigate the evolutionary process of the broccoli genome, we selected 13 representative Brassicaceae genomes, including A*. thaliana, B. carinata, B. napus, B. nigra, B. rapa*, and *B. olerace*a. The selected species were chosen based on their relevance to the broccoli genome and their representation of key phylogenetic lineages within the Brassicaceae family. These species include *A. thaliana*, a model species widely used in genome research; *B. carinata*, *B. napus*, and *B. nigra*, which represent important polyploid species; *B. rapa*, a close relative to broccoli; and *B. oleracea*, the species to which broccoli belongs. Specifically, the *B. oleracea* genomes include six distinct accessions (T08, T09, T11, T18, T27, and HDEM), which provide a broad genetic diversity for comparative analysis. The inclusion of *B. rapa* and *B. oleracea* genomes ensures that our analysis covers both the close relatives of broccoli and the broader evolutionary context within the Brassicaceae family. A phylogenetic tree of the 13 plant species was constructed using the RAXML package (v8.2.11) based on 32 906 high-quality single-copy orthologous genes. Divergence times were estimated using the MCMCTREE program (v1.0) with data from the TIMETREE database. The expansion and contraction of gene families were analyzed using CAFE (v3.1). To investigate polyploidization events in the Cichorium intybus genome, Ks (synonymous substitution rate) estimation was performed with KAKS_CALCULATOR (v2.0). Genome synteny comparisons and visualizations across multiple genomes were performed using the JCVI package (v1.1.23).

### SV analysis

The Nucmer program was used to align the SN60 genome to the NAU-LB assembly using the following parameters: mum -g 1000 -c 90 -l 40. Alignment block filtering was performed using a delta filter in a one-to-one alignment mode (−1). SNPs and InDels were extracted from the one-to-one alignment blocks using show-snp with the parameter setting ClrH from the MUMmer4 toolkit [42]. Functional effects of these variants were annotated using the SnpEff package. SVs, including translocations and inversions (≥1 Kb), were detected from nonallelic similarity blocks based on the resulting alignments, following previously reported methods using MUMmer4. Functional annotation of the SVs was performed using the ANNOVAR package.

### 3D-genomics analysis

First, clean Hi-C reads were mapped and processed using HIC-PRO software. The HIC-CONVERTFORMAT tool in the HIC-EXPLORER software package was used to generate Hi-C contact matrices at different resolutions. The HICPCA program was used to delineate the A/B compartments at a resolution of 50 kb. HICFINDTADs and HICMERGEDOMAINS were used to identify and merge topologically associated domain (TAD)-like structures, respectively. Hi-C interaction peaks were identified using the FIT-HI-C (v2.05) tool, with parameter settings of -r 10 000, -U 1000000, -L 20000, and-x intraOnly. Candidate interaction sites were identified using a false discovery rate (FDR) <0.00001 and contact count >10.

### Bulked segregant analysis

Genomic DNA from 30 individuals was pooled to create bulk samples with purple and light-green heads. Genomic DNA was extracted from the young leaves of the two bulk plants, along with that from two parental plants. These samples were used to construct paired-end (PE) sequencing libraries for next-generation sequencing (NGS). After sequencing, cleaned reads were aligned to the assembled genome of SN60 using BWA (v0.7.17-r1188) with the parameters “mem –M.” Variant calling was performed using GATK with the previously mentioned parameters. The resulting variants (SNPs and InDels) were annotated using SnpEff (v5.0). Association analysis was performed to identify genomic regions associated with head color using the SNP-index method and D(SNP-index) methods.

### RNA-seq analysis

RNA-seq analysis was conducted to identify DEGs between the BT126 and *bof3’h* mutant. Total RNA was extracted from the head buds. RNA-seq libraries were prepared using standard protocols followed by high-throughput sequencing. The preprocessing of raw reads involved quality control using tools such as FastQC to assess read quality and Trimmomatic for adapter removal and trimming of low-quality bases. Gene expression levels were quantified using HTSeq. Differential expression analysis was performed using the statistical tool DESeq2 [43]. The results were filtered based on a fold-change threshold and an adjusted *P*-value (e.g. FDR < 0.05) to prevent false positives. GO enrichment and KEGG pathway analyses were performed using ClusterProfiler to identify enriched biological processes and pathways associated with the phenotype of interest. Differential expression was determined using DESeq2 with FDR < 0.05. Three biological replicates per genotype were analyzed.

### Metabolome detection and analysis

The freeze-dried buds were ground into a fine powder using a mixer mill equipped with zirconia beads. A total of 100 mg of the powder was extracted with 1 mL of 70% aqueous methanol at 4°C overnight. The extract was centrifuged at 10000 × g for 10 min, and the supernatant was filtered through a 0.22-μm membrane for subsequent analysis. Separation was performed using a Waters ACQUITY UPLC HSS T3 C18 column (1.8 μm, 2.1 mm × 100 mm). Metabolite detection was conducted using an API 6500 Q TRAP mass spectrometer equipped with an ESI Turbo Ion-Spray interface in positive ion mode. Anthocyanin metabolites were detected and quantified in multiple reaction monitoring (MRM) mode. Metabolite identification was achieved by comparing the retention times and MS/MS fragment spectra with those of authentic standards. For data processing, multivariate statistical analyses (PCA, PLS-DA, and OPLS-DA) were performed to identify differential metabolites (VIP ≥ 1 and |log2foldchange| ≥ 1), and KEGG pathway enrichment analysis was utilized to further elucidate the relevant metabolic pathways and their biological significance.

To exclude the possibility that the absence of malvidin detection was caused by analytical limitations rather than biological differences, we performed a series of method validation assays. First, authentic malvidin-3-O-glucoside standards (Sigma-Aldrich) were analyzed under the same chromatographic and mass spectrometric conditions described above. The standard produced a distinct peak with high signal intensity (MRM transition: m/z 493 → 331) and a retention time of 3.42 min, confirming that the UPLC–MS settings were fully capable of detecting malvidin when present. Second, we examined both positive and negative ionization modes, as well as adjusted declustering potentials (DP), collision energies (CE), and collision cell exit potentials (CXP), but no malvidin-related ion signals were detected in any broccoli samples. Third, we extended the chromatographic gradient and broadened the retention time window to ensure that no late-eluting or co-eluting compounds interfered with malvidin detection. Collectively, these validation experiments demonstrate that the UPLC–MS method used in this study is sensitive and robust, and the absence of malvidin signal in SN60 samples reflects its true biological absence rather than technical limitations.

### Subcellular localization of BoF3’H and bof3’h

The CDS of *BoF3’H* and *bof3’h* were cloned from BT126 and SN60, respectively, and inserted into the ProCAMV35S vector. Constructs were introduced into Agrobacterium tumefaciens GV3101 and infiltrated into Nicotiana tabacum leaves (OD600 = 0.6–0.8). After 24 h in darkness and 24 h under light, GFP fluorescence was observed using a confocal laser scanning microscope (Leica Microsystems, Wetzlar, Germany) at 496–540 nm.

### Quantitative real-time PCR (qRT-PCR)

Total RNA was extracted from flowers, leaves, roots, buds, and stems of BT126 and SN60 plants. First-strand cDNA was synthesized from 1 μg of total RNA using a reverse transcription kit following the manufacturer’s instructions. Quantitative real-time PCR (qRT-PCR) was carried out on a QuantStudio™ 6 Real-Time PCR System (Applied Biosystems, USA) using Ultra SYBR Green Mix (Kangwei Century, Beijing, China). The Actin gene was employed as an internal reference. Relative transcript abundance was determined using the 2^–ΔΔCt^ method. Each qRT-PCR reaction included three biological replicates, with three technical replicates per biological sample. Statistical significance of expression differences was assessed using Student’s *t*-test.

### Genetic transformation and CRISPR/Cas9-mediated gene editing in broccoli

The sequence of *BoF3’H* found in broccoli BT126 was used for sgRNA design. Two sgRNA sequences targeting the first exon were selected and inserted into a modified vector under control of the Arabidopsis U6 promoter. Then, this construct was cloned into a vector containing the CaMV 35S promoter, which drives both the bar selection marker and the Cas9 gene, as described in an earlier study. The Agrobacterium-mediated transformation of broccoli BT126 was performed according to previously established protocols. Transgenic plants were selected based on resistance to Basta, which indicated successful integration of the bar gene and the CRISPR/Cas9 construct. To verify the success of genome editing, we amplified the genomic regions flanking the sgRNA-targeted sites from positive transgenic plants and subjected them to Sanger sequencing. To assess mutations in the T0 generation, we cloned and sequenced the PCR products.

### Expression and promoter analysis

Promoter regions (2 kb upstream of the transcription start site) of *BoF3’H* from BT126 were amplified using high-fidelity PCR and Sanger sequenced. Promoter sequences of *Bol043829*, *BnaC09G0570900ZS*, *BraA10g02723P*, *BnaA10G0256900ZS,* and *BoF3’H*-BT126 were aligned using MUSCLE, and polymorphisms were manually inspected. Cis-regulatory elements were annotated using PlantCARE with default thresholds. MYC, G-box, and ERE motifs were further verified through motif-scanning with FIMO (MEME Suite). The presence/absence and structural differences of cis-elements were compared between genotypes to evaluate their potential regulatory influence on *BoF3’H* expression.

### Statistical analysis

All statistical analyses were performed using R (v4.2.0) and GraphPad Prism (v9.0). For qRT-PCR, metabolite quantification, and phenotypic measurements, three biological replicates were used unless otherwise stated, each with three technical replicates. Data are presented as mean ± SD. Statistical significance was assessed using two-tailed Student’s *t*-tests for pairwise comparisons or one-way ANOVA followed by Tukey’s post hoc test for multiple comparisons. Significance thresholds were set as *P* < 0.05, *P* < 0.01, and *P* < 0.001. For RNA-seq data, differentially expressed genes were identified using DESeq2 with FDR < 0.05 (Benjamini–Hochberg correction). For metabolomic analysis, differential metabolites were defined by VIP ≥ 1 and |log2-fold change| ≥ 1. Error bars shown in all figures represent standard deviation unless indicated otherwise.

## Acknowledgments

This study was supported by the Shanghai Agricultural Science and Technology Innovation Program (grant no. K2023008) and the Excellent Team Project (Nongkezhuo 2022(007)).

## Supplementary Material

Web_Material_uhag110

## Data Availability

All raw sequencing data (including long-read, short-read, and Hi-C data) and the final T2T genome assembly have been deposited in the Genome Sequence Archive (GSA) of the China National Center for Bioinformation (https://www.cncb.ac.cn/) under accession number CRA033836, within BioProject PRJCA051561.
