# Supplementary material for: T2T reference genome assembly provides insights into anthocyanin accumulation in broccoli
Source: Hortic Res. 2026 Apr 21;13(7):uhag110. doi: 10.1093/hr/uhag110 (PMC13305774; doi:10.1093/hr/uhag110)
Supplement: Web_Material_uhag110 [file web_material_uhag110.zip › Figure S1-S14.docx]

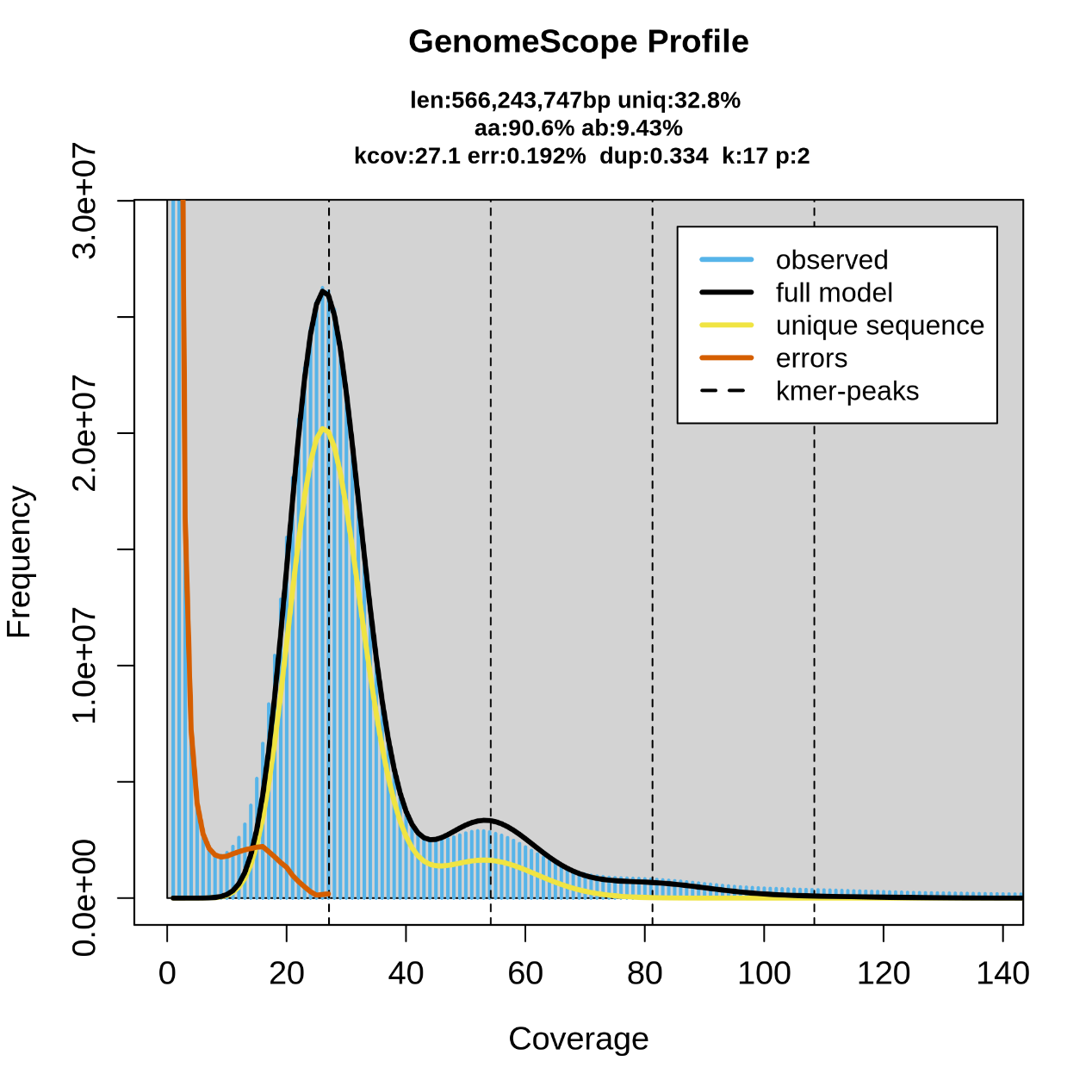


**Figure S1** Genome Survey result of 21B15


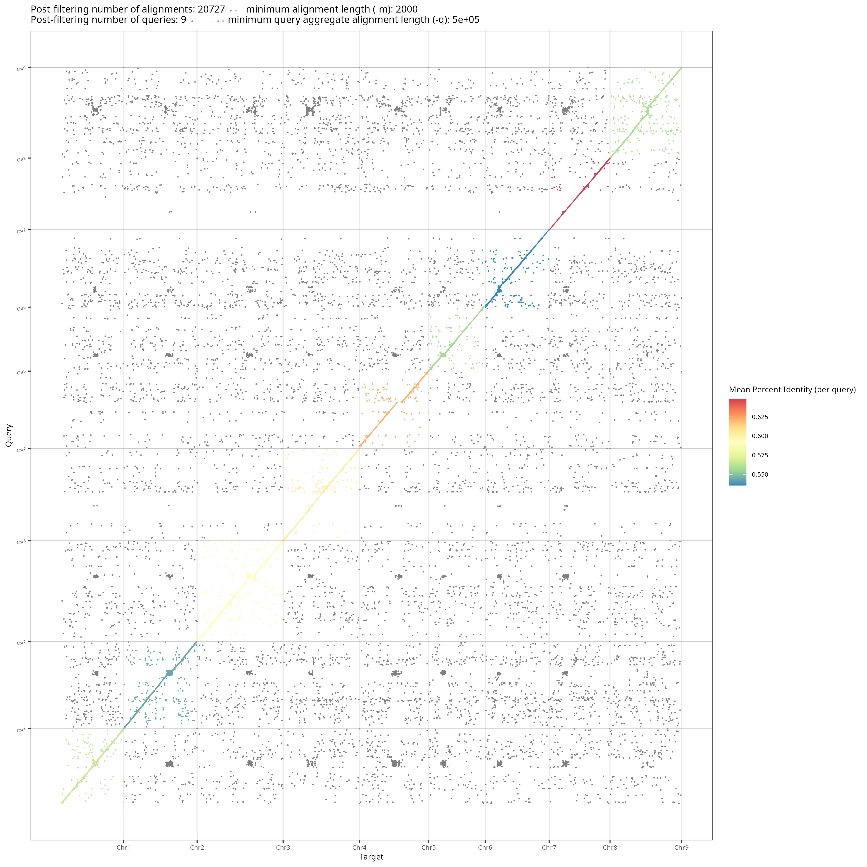


**Figure S2** Collinearity of the genomes of 21B15 and BOP04-28-6


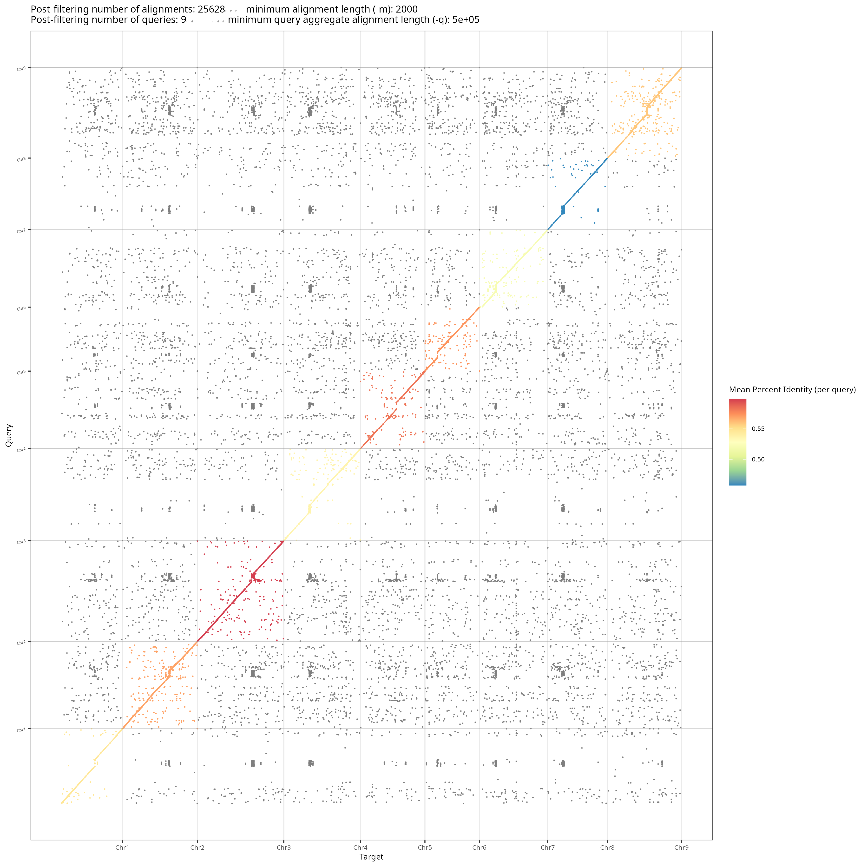


**Figure S3** Collinearity of the genomes of 21B15 and HDEM


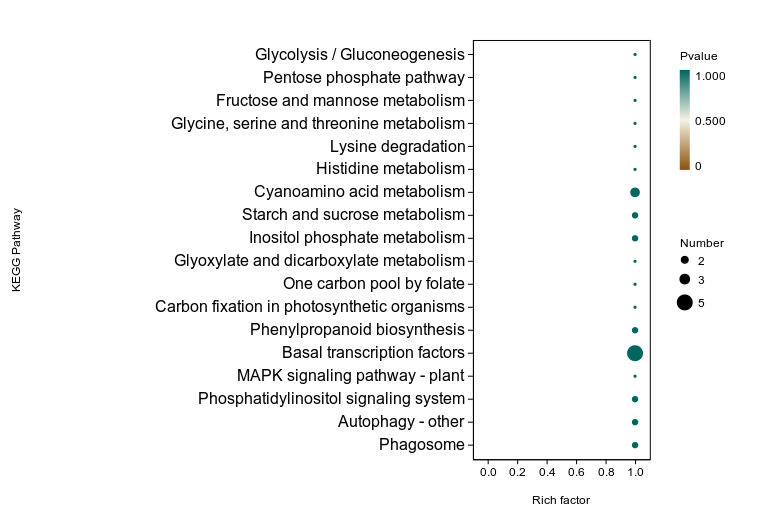


**Figure S4** Functional annotation of genes in the centromere region


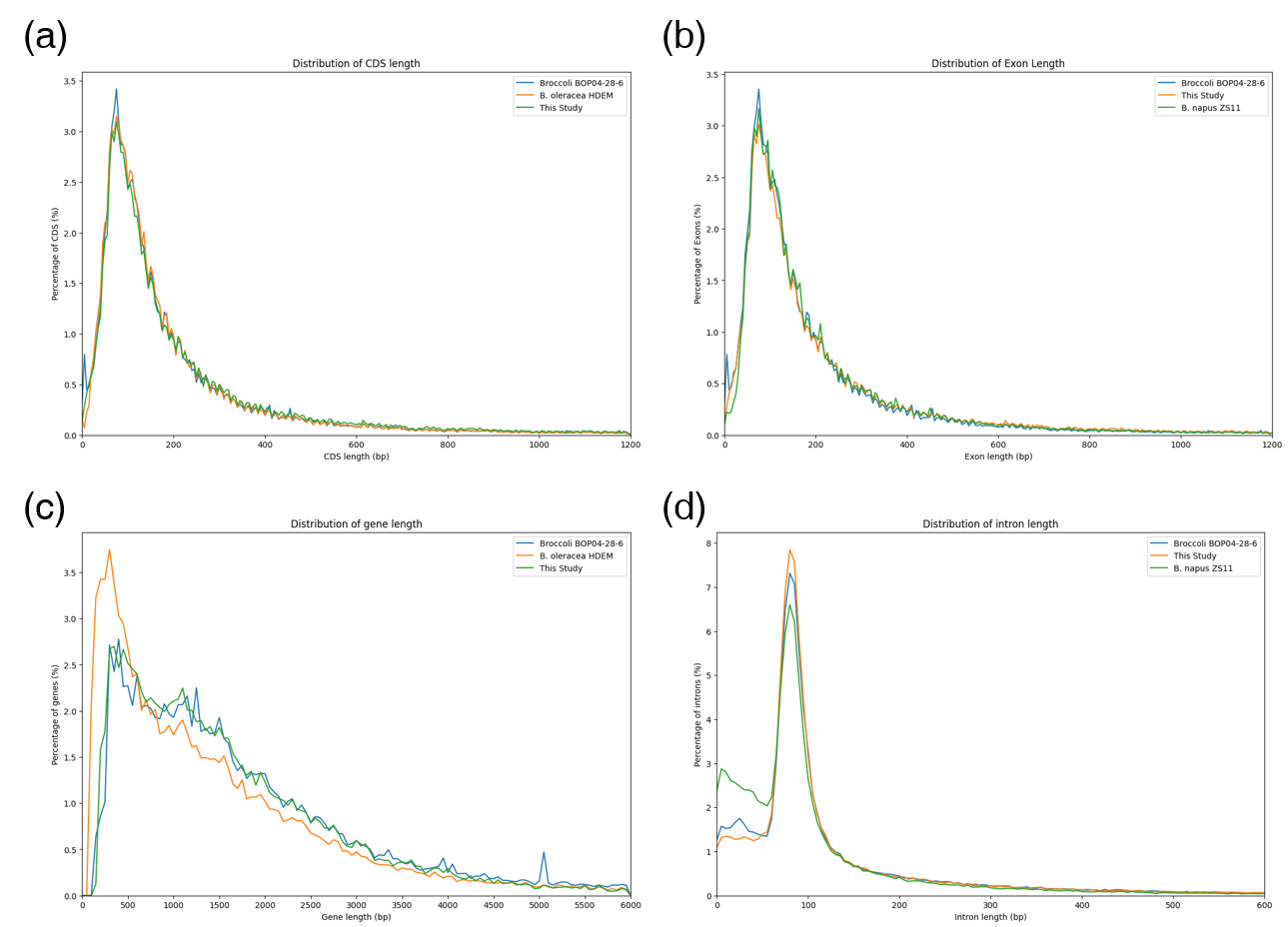


**Figure S5** Distribution of the length of gene and gene components in the 21B15 assembly. (a) Coding sequence (CDS) length. (b) Exon length. (c) Gene length. (d) Intron length. The y-axis represents the percentage of CDS, exons, genes and introns with a certain length.


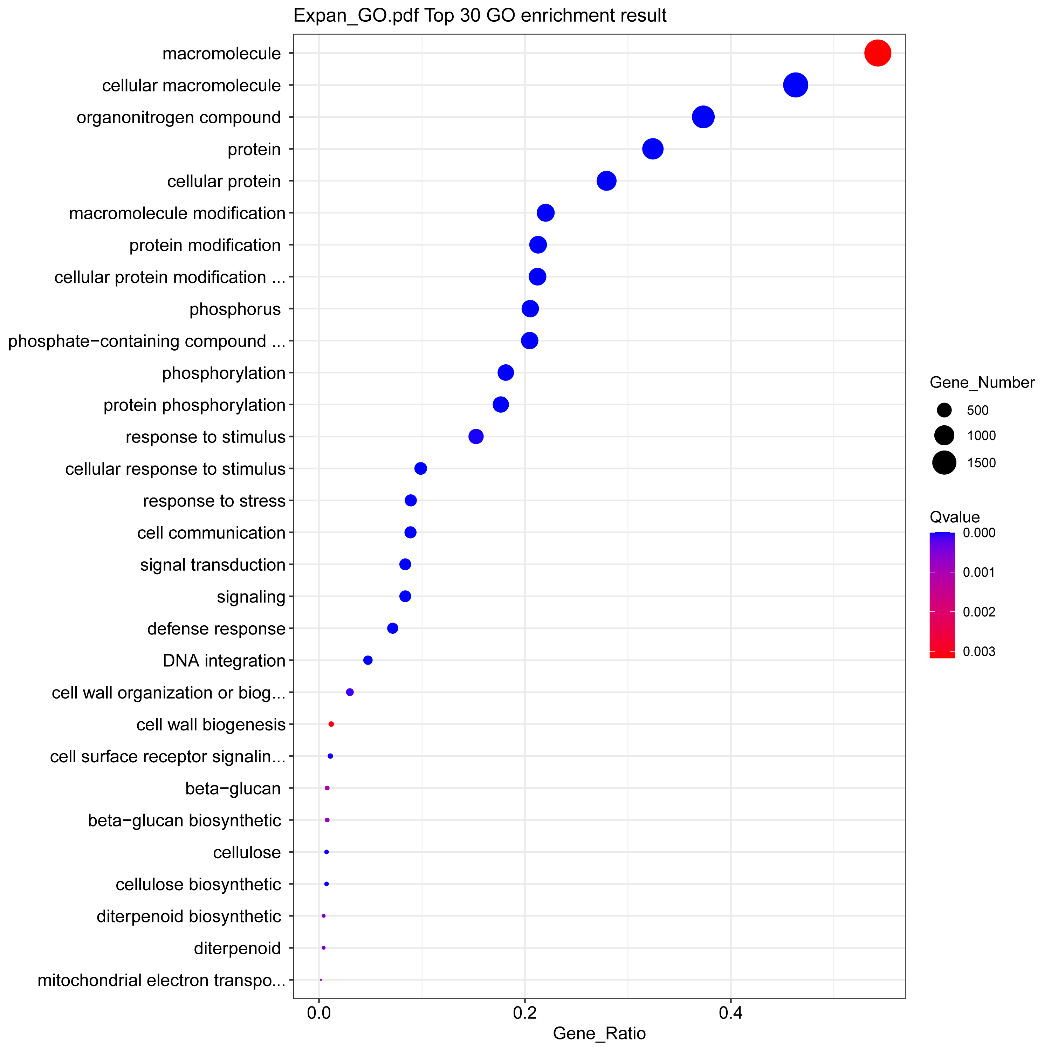


**Figure S6** Significantly expanded gene family (P < 0.05) GO enrichment bubble plot


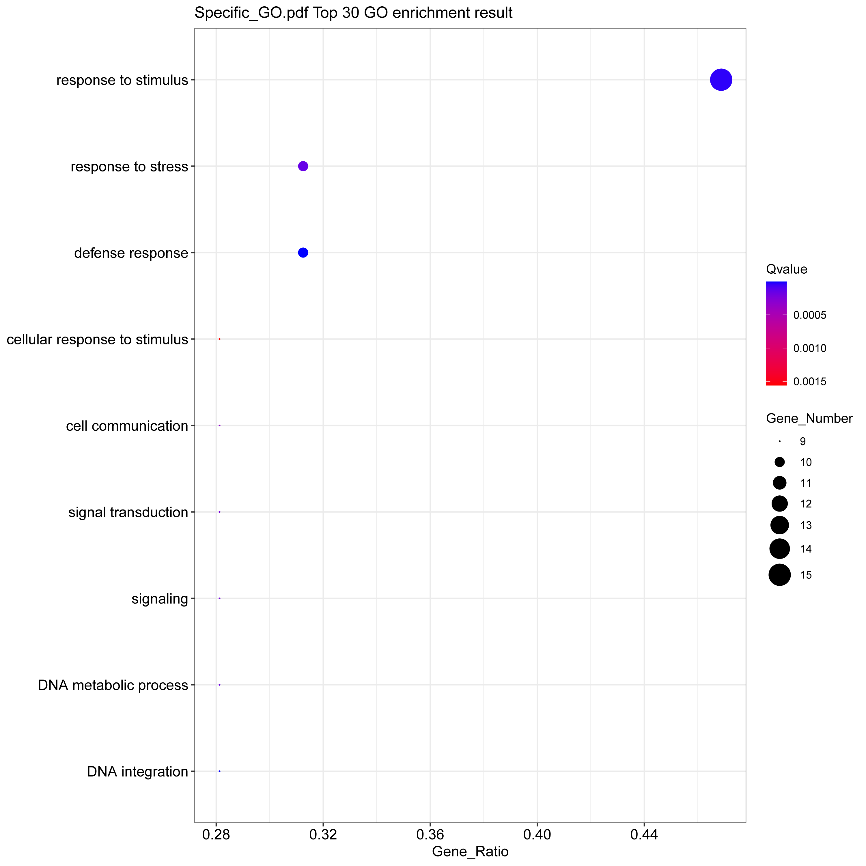


**Figure S7** Significantly specific gene family (P < 0.05) GO enrichment bubble plot


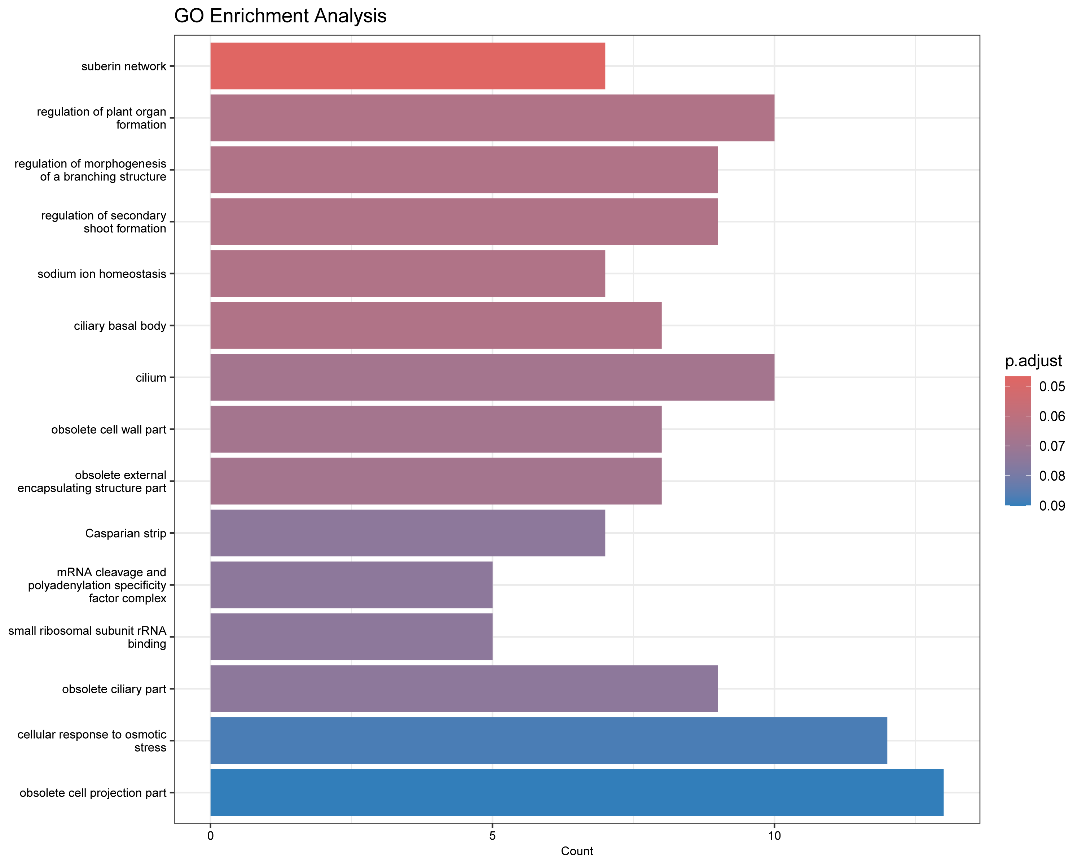


**Figure S8** GO enrichment of the SNP/indel-genes between 21B15 and BOP04-28-6


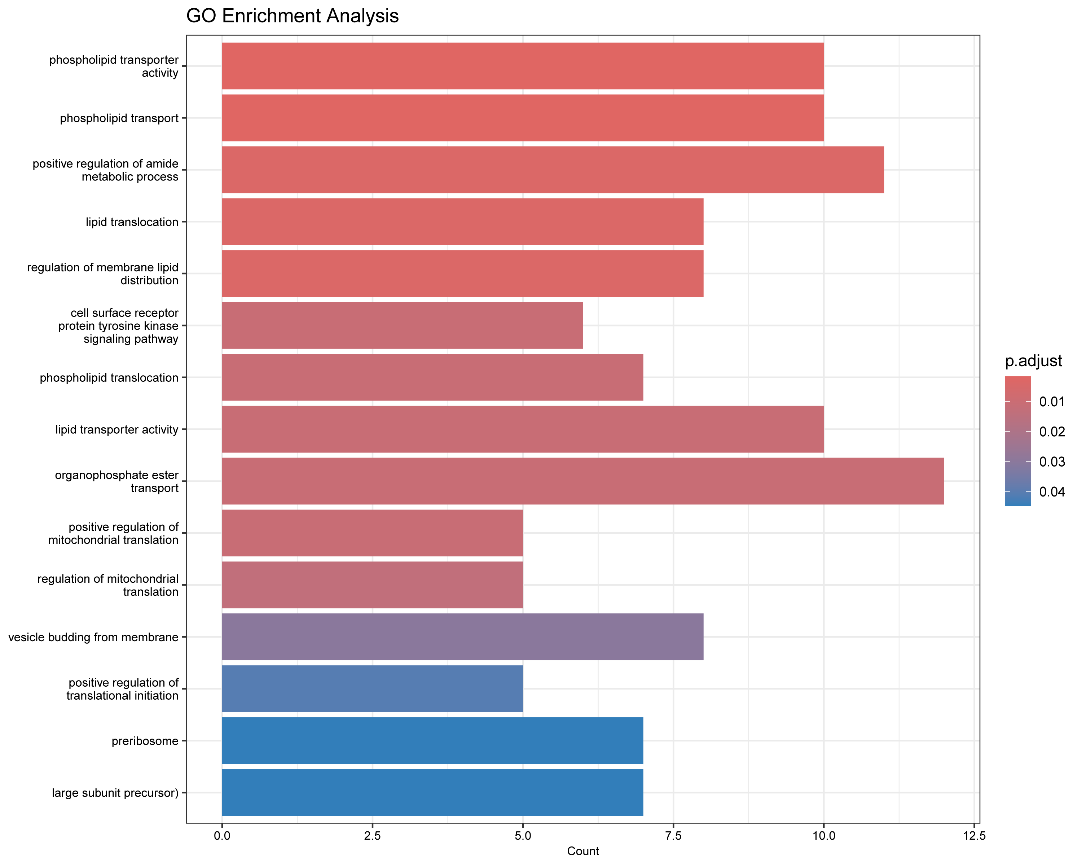


**Figure S9** GO enrichment of the SV-genes between 21B15 and BOP04-28-6


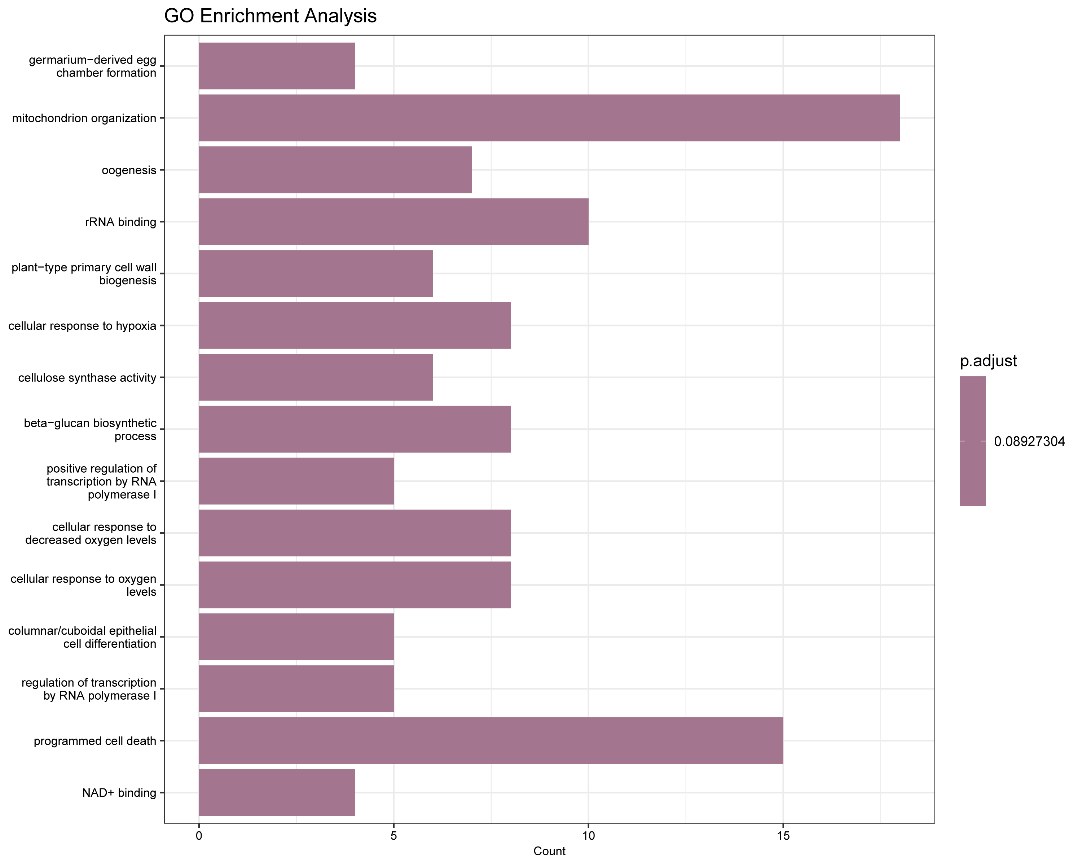


**Figure S10** GO enrichment of the SNP/indel-genes between 21B15 and HDEM


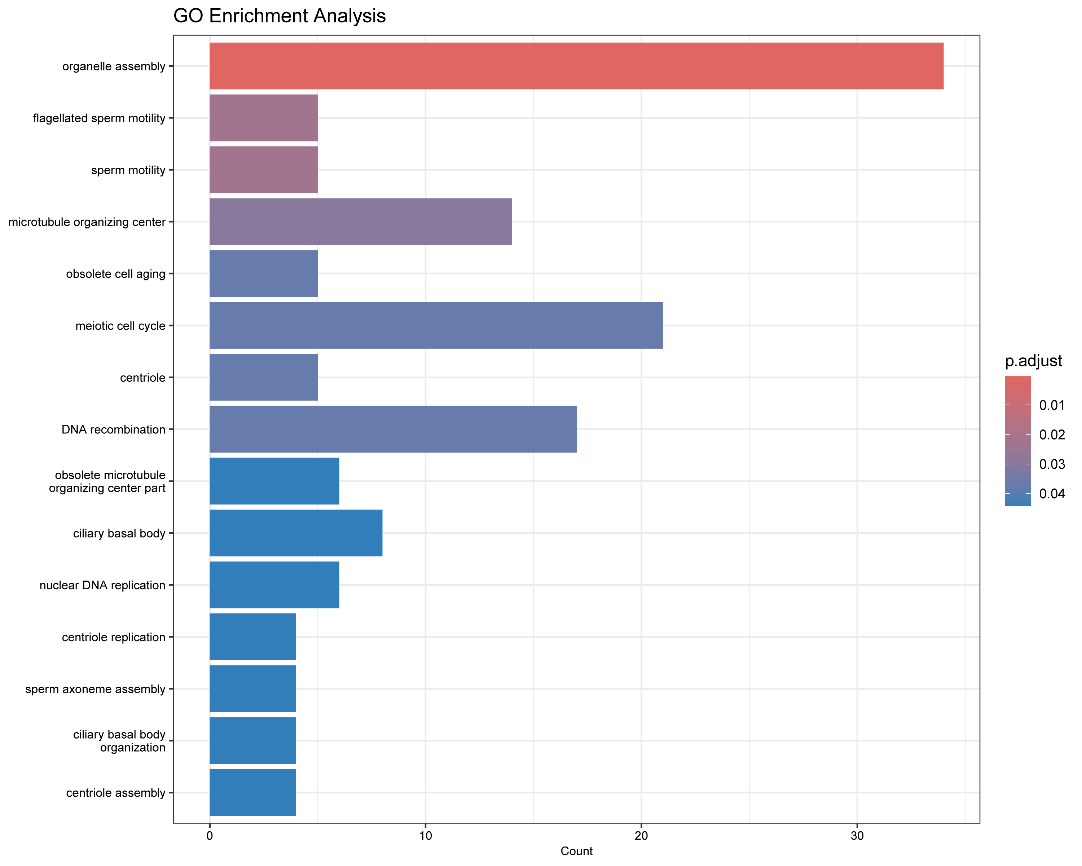


**Figure S11** GO enrichment of the SV-genes between 21B15 and HDEM


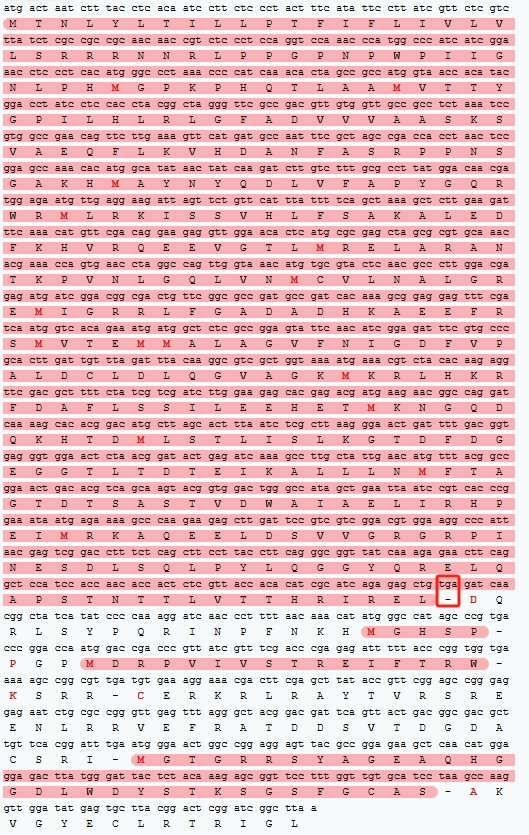


**Figure S12** The alignment of partial cDNA and predicted amino sequences of the F3′H gene in SN60. A 43-bp deletion was identified sequence and this deletion results in an early appearance of the stop codon (TGA)


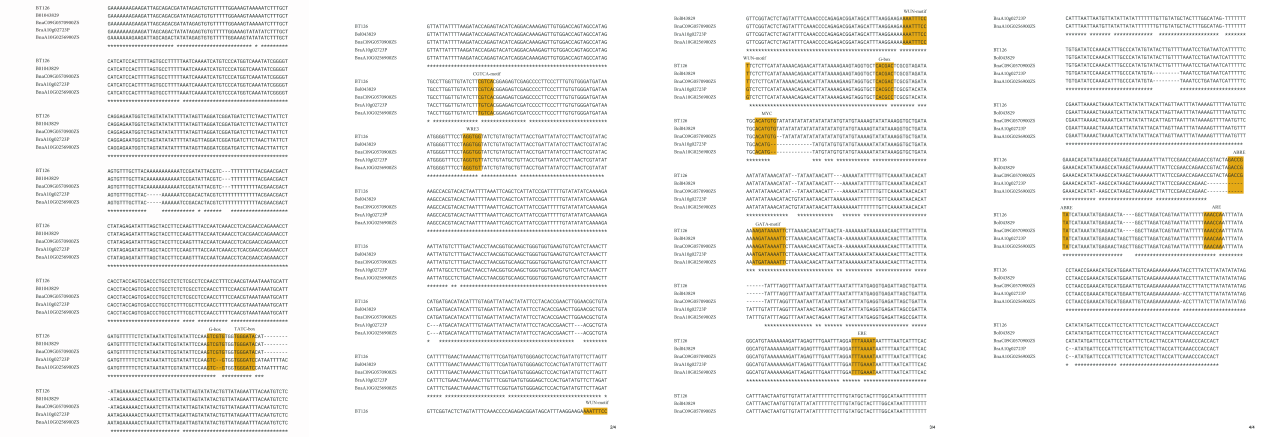


**Figure S13** Promoter Cis-regulatory elements analysis of *Bol043829*, BnaC09G0570900ZS, BraA10g02723P, *BnaA10G0256900ZS* and *BoF3’H-BT126* genes.


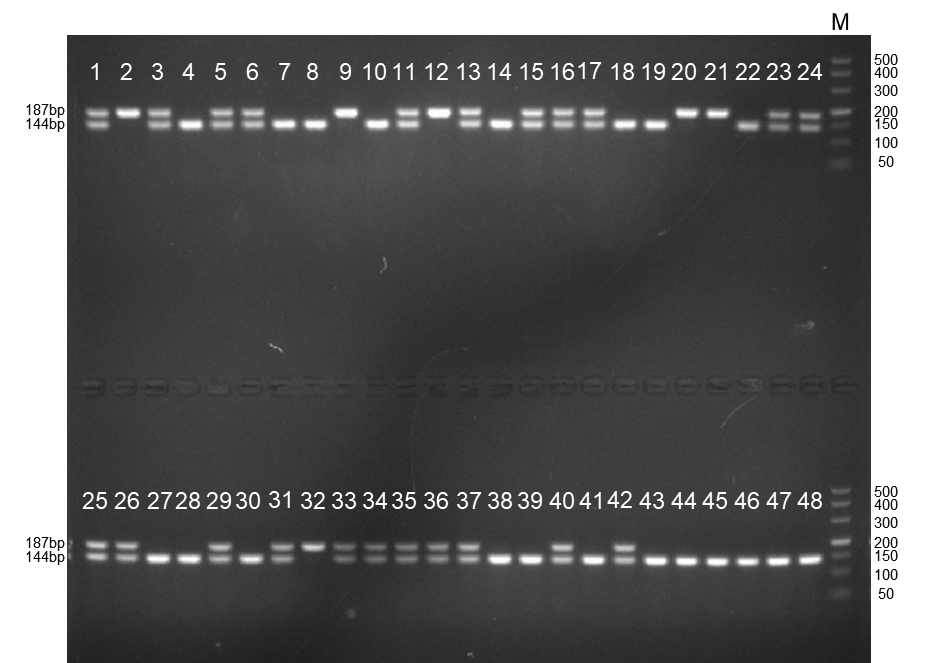


**Figure S14** PCR amplification patterns of the *BoF3’H* InDel marker in representative F₂ individuals. Green-curd plants (4,7,8,10,14,18,19,22,27,28,30,38,39,41,43,44,45,46,47,48) exhibited only the 144 bp fragment, whereas purple-curd plants (1,2,3,5,6,9,11,12,13,15,16,17,20,21,23,24,25,26,29,31,32,33,34,35,36,37,40,42) showed either a 187 bp fragment or both 187 bp and 144 bp fragments.
